# 10-Allyl-2,3-dihydro-1*H*-pyrrolo[2,1-*c*][1,4]benzodiazepine-5,11(10*H*,11a*H*)-dione

**DOI:** 10.1107/S1600536809034266

**Published:** 2009-09-05

**Authors:** Hanane Benzeid, El Mokhtar Essassi, Nathalie Saffon, Bernard Garrigues, Seik Weng Ng

**Affiliations:** a Laboratoire de Chimie Organique Hétérocyclique, Pôle de compétences Pharmacochimie, Université Mohammed V-Agdal, BP 1014 Avenue Ibn Batout, Rabat, Morocco; b Service commun Rayons X, Université Paul Sabatier, Bâtiment 2R1, 118 route de Narbonne, 31062 Toulouse, France; c Hétérochimie Fondamentale et Appliquée, Université Paul Sabatier, UMR 5069, 118 Route de Narbonne, 31062 Toulouse, France; d Department of Chemistry, University of Malaya, 50603 Kuala Lumpur, Malaysia

## Abstract

The compound, C_15_H_16_N_2_O_2_, features a pyrroline ring fused with a seven-membered diazepine ring; the latter system adopts a boat conformation (with the methine C atom as the prow and the two C atoms of the aromatic ring as the stern). A CH_2_–CH_2_ segment of the pyrroline ring is disordered over two positions in a 1:1 ratio.

## Related literature

Pyrrolo[2,1-*c*][1,4]benzodiazepines are potent, naturally occurring anti­tumor anti­biotics produced by *Streptomyces *species; see: Cargill *et al.* (1974 ▶); Thurston *et al.* (1993 ▶). For the design and synthesis of DNA inter-strand cross-linking as well as conjugate agents to enhance the sequence selectivity and to increase selectivity for tumor cells, see: Bose *et al.* (1992 ▶); Gregson *et al.* (2004 ▶).
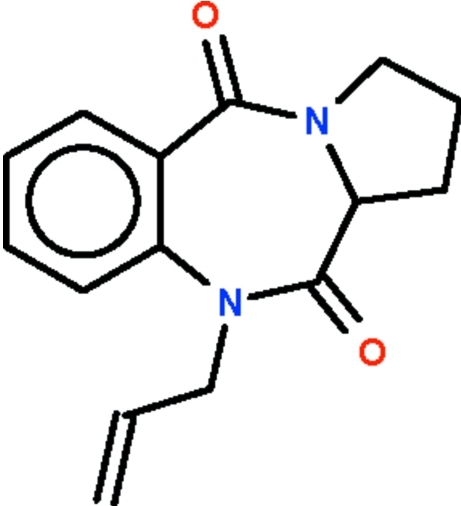


## Experimental

### 

#### Crystal data

C_15_H_16_N_2_O_2_
*M* *_r_* = 256.30
Orthorhombic, 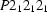

*a* = 7.0988 (1) Å
*b* = 11.7166 (2) Å
*c* = 15.6592 (3) Å
*V* = 1302.44 (4) Å^3^
*Z* = 4
Mo *K*α radiation
μ = 0.09 mm^−1^
*T* = 193 K
0.30 × 0.30 × 0.20 mm

#### Data collection

Bruker APEXII diffractometer
Absorption correction: none
20329 measured reflections
2263 independent reflections
1900 reflections with *I* > 2σ(*I*)
*R*_int_ = 0.031

#### Refinement

*R*[*F* ^2^ > 2σ(*F* ^2^)] = 0.049
*wR*(*F* ^2^) = 0.146
*S* = 1.04
2263 reflections
178 parameters
15 restraints
H-atom parameters constrained
Δρ_max_ = 0.53 e Å^−3^
Δρ_min_ = −0.30 e Å^−3^

### 

Data collection: *APEX2* (Bruker, 2005 ▶); cell refinement: *SAINT* (Bruker, 2005 ▶); data reduction: *SAINT*; program(s) used to solve structure: *SHELXS97* (Sheldrick, 2008 ▶); program(s) used to refine structure: *SHELXL97* (Sheldrick, 2008 ▶); molecular graphics: *X-SEED* (Barbour, 2001 ▶); software used to prepare material for publication: *publCIF* (Westrip, 2009 ▶).

## Supplementary Material

Crystal structure: contains datablocks global, I. DOI: 10.1107/S1600536809034266/sj2637sup1.cif

Structure factors: contains datablocks I. DOI: 10.1107/S1600536809034266/sj2637Isup2.hkl

Additional supplementary materials:  crystallographic information; 3D view; checkCIF report

## Figures and Tables

**Fig. 1.**
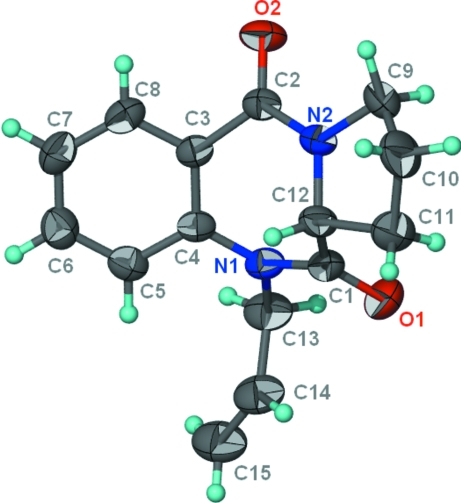
Thermal ellipsoid plot (Barbour, 2001) of C15H16N2O2 at the 70% probability level; hydrogen atoms are drawn as spheres of arbitrary radius.

## supplementary crystallographic information

### Experimental

2,3-Dihydro-*1H*-pyrrolo[2,1-*c*][1,4]benzodiazepine-5,11(10*H*,11*a*H)-dione (1 g, 4.6 mmol), allyl bromide (0.64 g, 4.6 mmol) and potassium carbonate (0.64 g, 4.6 mmol) along with a catalytic amount of tetra-*n*-butyammonium bromide were stirred in *N*,*N*-dimethylformamide (20 ml) for 12 h. After the completion of the reaction (as monitored by TLC), the solid material was removed by filtration and the solvent evaporated under vacuum. Dichloromethane (20 ml) was added and the solution filtered. The solvent was removed and the product purified by recrystallization from dichloromethane to afford colorless crystals in 80% yield. The formulation was established by proton and carbon-13 NMR spectroscopy in CDCl_3_.

### Refinement

Carbon-bound H-atoms were placed in calculated positions (C—H 0.95 to 0.99 Å) and were included in the refinement in the riding model approximation, with *U*(H) set to 1.2*U*(C).

Two of the carbon atoms in the tetrahydropyrrolyl ring are disordered over two positions; the occupancy cound not be refined, and was assumed to be 50:50. The pairs of carbon-carbon (C10–C11, C10'–C11') distances were restrained to within 0.01 Å of each other, and the temperature factors of the primed atoms were restrained to those of the unprimed ones. Their anisotropic temperature factors were restrained to nearly isotropic values.

In the absence of significant anomalous dispersion effects, Freidel pairs were merged.

### Crystal data

**Table:**

C_15_H_16_N_2_O_2_	*F*(000) = 544
*M**_r_* = 256.30	*D*_x_ = 1.307 Mg m^−^^3^
Orthorhombic, *P*2_1_2_1_2_1_	Mo *K*α radiation, λ = 0.71073 Å
Hall symbol: P 2ac 2ab	Cell parameters from 7433 reflections
*a* = 7.0988 (1) Å	θ = 2.2–25.3°
*b* = 11.7166 (2) Å	µ = 0.09 mm^−^^1^
*c* = 15.6592 (3) Å	*T* = 193 K
*V* = 1302.44 (4) Å^3^	Block, colorless
*Z* = 4	0.30 × 0.30 × 0.20 mm

### Data collection

**Table:**

Bruker APEXII diffractometer	1900 reflections with *I* > 2σ(*I*)
Radiation source: fine-focus sealed tube	*R*_int_ = 0.031
graphite	θ_max_ = 30.5°, θ_min_ = 5.2°
φ and ω scans	*h* = −10→10
20329 measured reflections	*k* = −16→14
2263 independent reflections	*l* = −22→19

### Refinement

**Table:**

Refinement on *F*^2^	Primary atom site location: structure-invariant direct methods
Least-squares matrix: full	Secondary atom site location: difference Fourier map
*R*[*F*^2^ > 2σ(*F*^2^)] = 0.049	Hydrogen site location: inferred from neighbouring sites
*wR*(*F*^2^) = 0.146	H-atom parameters constrained
*S* = 1.03	*w* = 1/[σ^2^(*F*_o_^2^) + (0.086*P*)^2^ + 0.2922*P*] where *P* = (*F*_o_^2^ + 2*F*_c_^2^)/3
2263 reflections	(Δ/σ)_max_ = 0.001
178 parameters	Δρ_max_ = 0.53 e Å^−^^3^
15 restraints	Δρ_min_ = −0.30 e Å^−^^3^

### Fractional atomic coordinates and isotropic or equivalent isotropic displacement parameters (Å^2^)

**Table:**

	*x*	*y*	*z*	*U*_iso_*/*U*_eq_	Occ. (<1)
O1	0.3349 (3)	0.89582 (17)	0.62000 (12)	0.0545 (5)	
O2	−0.1387 (3)	0.62142 (13)	0.76109 (11)	0.0423 (4)	
N1	0.2785 (2)	0.86934 (15)	0.76059 (12)	0.0312 (4)	
N2	−0.0624 (3)	0.77145 (15)	0.67775 (12)	0.0327 (4)	
C1	0.2213 (4)	0.88657 (18)	0.67841 (15)	0.0355 (5)	
C2	−0.0695 (3)	0.71719 (17)	0.75353 (14)	0.0301 (4)	
C3	−0.0008 (3)	0.78280 (17)	0.82960 (13)	0.0280 (4)	
C4	0.1542 (3)	0.85792 (16)	0.83096 (13)	0.0279 (4)	
C5	0.1940 (3)	0.91758 (17)	0.90646 (13)	0.0333 (4)	
H5	0.2989	0.9678	0.9083	0.040*	
C6	0.0828 (4)	0.9044 (2)	0.97834 (14)	0.0396 (5)	
H6	0.1090	0.9476	1.0283	0.048*	
C7	−0.0675 (4)	0.8278 (2)	0.97772 (14)	0.0407 (5)	
H7	−0.1425	0.8172	1.0274	0.049*	
C8	−0.1059 (3)	0.76768 (19)	0.90409 (14)	0.0359 (5)	
H8	−0.2069	0.7144	0.9040	0.043*	
C9	−0.1524 (4)	0.7262 (2)	0.60032 (14)	0.0399 (5)	
H9A	−0.2678	0.6823	0.6137	0.048*	0.50
H9B	−0.0651	0.6780	0.5667	0.048*	0.50
H9C	−0.2759	0.6916	0.6143	0.048*	0.50
H9D	−0.0719	0.6671	0.5736	0.048*	0.50
C10	−0.197 (3)	0.8367 (11)	0.5551 (12)	0.059 (3)	0.50
H10A	−0.2187	0.8236	0.4935	0.071*	0.50
H10B	−0.3102	0.8732	0.5800	0.071*	0.50
C11	−0.025 (2)	0.9097 (17)	0.5692 (5)	0.047 (2)	0.50
H11A	−0.0500	0.9912	0.5570	0.056*	0.50
H11B	0.0832	0.8835	0.5342	0.056*	0.50
C10'	−0.178 (3)	0.8258 (11)	0.5408 (12)	0.059 (3)	0.50
H10C	−0.3124	0.8465	0.5366	0.071*	0.50
H10D	−0.1312	0.8061	0.4830	0.071*	0.50
C11'	−0.066 (2)	0.9241 (16)	0.5769 (5)	0.047 (2)	0.50
H11C	−0.1475	0.9923	0.5823	0.056*	0.50
H11D	0.0400	0.9430	0.5384	0.056*	0.50
C12	0.0096 (3)	0.88869 (18)	0.66551 (14)	0.0339 (5)	
H12	−0.0551	0.9459	0.7027	0.041*	0.50
H12'	−0.0479	0.9394	0.7098	0.041*	0.50
C13	0.4837 (3)	0.8775 (2)	0.77691 (19)	0.0433 (6)	
H13A	0.5522	0.8322	0.7335	0.052*	
H13B	0.5121	0.8446	0.8337	0.052*	
C14	0.5520 (4)	1.0005 (2)	0.7740 (2)	0.0490 (6)	
H14	0.5020	1.0490	0.7309	0.059*	
C15	0.6751 (4)	1.0438 (3)	0.8269 (2)	0.0565 (7)	
H15A	0.7278	0.9976	0.8707	0.068*	
H15B	0.7119	1.1214	0.8216	0.068*	

### Atomic displacement parameters (Å^2^)

**Table:**

	*U*^11^	*U*^22^	*U*^33^	*U*^12^	*U*^13^	*U*^23^
O1	0.0621 (12)	0.0550 (11)	0.0466 (10)	−0.0140 (10)	0.0231 (9)	−0.0031 (9)
O2	0.0473 (9)	0.0272 (7)	0.0525 (10)	−0.0097 (7)	−0.0042 (8)	0.0039 (7)
N1	0.0253 (7)	0.0269 (8)	0.0413 (10)	−0.0014 (6)	0.0036 (7)	0.0012 (7)
N2	0.0361 (8)	0.0276 (8)	0.0345 (8)	−0.0072 (7)	−0.0013 (8)	−0.0020 (7)
C1	0.0425 (11)	0.0278 (9)	0.0362 (10)	−0.0075 (9)	0.0072 (9)	−0.0012 (8)
C2	0.0273 (8)	0.0252 (8)	0.0380 (10)	−0.0003 (7)	0.0003 (9)	0.0007 (8)
C3	0.0266 (8)	0.0245 (8)	0.0331 (9)	0.0012 (7)	−0.0001 (8)	0.0042 (7)
C4	0.0267 (8)	0.0225 (8)	0.0345 (9)	0.0010 (7)	−0.0026 (8)	0.0043 (7)
C5	0.0358 (10)	0.0277 (9)	0.0362 (10)	−0.0004 (8)	−0.0083 (9)	0.0034 (8)
C6	0.0487 (13)	0.0388 (11)	0.0312 (10)	0.0040 (10)	−0.0062 (10)	0.0022 (9)
C7	0.0467 (12)	0.0436 (12)	0.0320 (10)	0.0029 (11)	0.0042 (10)	0.0098 (9)
C8	0.0345 (10)	0.0360 (10)	0.0372 (10)	−0.0027 (9)	0.0015 (9)	0.0095 (9)
C9	0.0446 (12)	0.0394 (11)	0.0357 (11)	−0.0074 (10)	−0.0010 (10)	−0.0097 (9)
C10	0.088 (4)	0.059 (3)	0.031 (5)	−0.015 (3)	−0.015 (4)	0.000 (3)
C11	0.061 (6)	0.042 (4)	0.0371 (16)	−0.008 (4)	−0.012 (3)	0.010 (2)
C10'	0.088 (4)	0.059 (3)	0.031 (5)	−0.015 (3)	−0.015 (4)	0.000 (3)
C11'	0.061 (6)	0.042 (4)	0.0371 (16)	−0.008 (4)	−0.012 (3)	0.010 (2)
C12	0.0440 (11)	0.0259 (9)	0.0319 (10)	−0.0067 (9)	−0.0066 (9)	0.0041 (8)
C13	0.0253 (9)	0.0403 (12)	0.0643 (16)	0.0012 (9)	0.0044 (10)	−0.0007 (11)
C14	0.0315 (11)	0.0506 (14)	0.0648 (16)	−0.0065 (11)	0.0037 (12)	0.0040 (13)
C15	0.0413 (13)	0.0502 (14)	0.078 (2)	−0.0061 (12)	0.0002 (15)	−0.0023 (14)

### Geometric parameters (Å, °)

**Table:**

O1—C1	1.224 (3)	C9—H9C	0.9900
O2—C2	1.231 (3)	C9—H9D	0.9900
N1—C1	1.365 (3)	C10—C11	1.509 (9)
N1—C4	1.418 (3)	C10—H10A	0.9900
N1—C13	1.482 (3)	C10—H10B	0.9900
N2—C2	1.347 (3)	C11—C12	1.547 (6)
N2—C9	1.470 (3)	C11—H11A	0.9900
N2—C12	1.478 (3)	C11—H11B	0.9900
C1—C12	1.517 (4)	C10'—C11'	1.507 (9)
C2—C3	1.499 (3)	C10'—H10C	0.9900
C3—C8	1.396 (3)	C10'—H10D	0.9900
C3—C4	1.409 (3)	C11'—C12	1.545 (6)
C4—C5	1.402 (3)	C11'—H11C	0.9900
C5—C6	1.383 (3)	C11'—H11D	0.9900
C5—H5	0.9500	C12—H12	1.0000
C6—C7	1.395 (4)	C12—H12'	1.0000
C6—H6	0.9500	C13—C14	1.522 (3)
C7—C8	1.378 (3)	C13—H13A	0.9900
C7—H7	0.9500	C13—H13B	0.9900
C8—H8	0.9500	C14—C15	1.306 (4)
C9—C10'	1.504 (7)	C14—H14	0.9500
C9—C10	1.510 (7)	C15—H15A	0.9500
C9—H9A	0.9900	C15—H15B	0.9500
C9—H9B	0.9900		
			
C1—N1—C4	124.17 (18)	C11—C10—H10A	110.9
C1—N1—C13	116.5 (2)	C9—C10—H10A	110.9
C4—N1—C13	118.9 (2)	C11—C10—H10B	110.9
C2—N2—C9	122.70 (18)	C9—C10—H10B	110.9
C2—N2—C12	124.44 (18)	H10A—C10—H10B	108.9
C9—N2—C12	112.27 (18)	C10—C11—C12	100.3 (12)
O1—C1—N1	121.5 (2)	C10—C11—H11A	111.7
O1—C1—C12	123.5 (2)	C12—C11—H11A	111.7
N1—C1—C12	115.02 (19)	C10—C11—H11B	111.7
O2—C2—N2	122.0 (2)	C12—C11—H11B	111.7
O2—C2—C3	121.4 (2)	H11A—C11—H11B	109.5
N2—C2—C3	116.48 (18)	C9—C10'—C11'	107.4 (12)
C8—C3—C4	118.96 (19)	C9—C10'—H10C	110.2
C8—C3—C2	115.13 (18)	C11'—C10'—H10C	110.2
C4—C3—C2	125.89 (18)	C9—C10'—H10D	110.2
C5—C4—C3	118.78 (19)	C11'—C10'—H10D	110.2
C5—C4—N1	118.87 (18)	H10C—C10'—H10D	108.5
C3—C4—N1	122.21 (18)	C10'—C11'—C12	108.3 (12)
C6—C5—C4	121.0 (2)	C10'—C11'—H11C	110.0
C6—C5—H5	119.5	C12—C11'—H11C	110.0
C4—C5—H5	119.5	C10'—C11'—H11D	110.0
C5—C6—C7	120.2 (2)	C12—C11'—H11D	110.0
C5—C6—H6	119.9	H11C—C11'—H11D	108.4
C7—C6—H6	119.9	N2—C12—C1	108.08 (19)
C8—C7—C6	119.1 (2)	N2—C12—C11'	104.2 (8)
C8—C7—H7	120.5	C1—C12—C11'	117.9 (7)
C6—C7—H7	120.5	N2—C12—C11	102.7 (8)
C7—C8—C3	121.9 (2)	C1—C12—C11	106.7 (6)
C7—C8—H8	119.1	N2—C12—H12	112.9
C3—C8—H8	119.1	C1—C12—H12	112.9
N2—C9—C10'	106.4 (8)	C11'—C12—H12	100.6
N2—C9—C10	99.7 (8)	C11—C12—H12	112.9
N2—C9—H9A	111.8	N2—C12—H12'	108.8
C10'—C9—H9A	115.9	C1—C12—H12'	108.8
C10—C9—H9A	111.8	C11'—C12—H12'	108.8
N2—C9—H9B	111.8	C11—C12—H12'	121.1
C10'—C9—H9B	100.8	N1—C13—C14	111.7 (2)
C10—C9—H9B	111.8	N1—C13—H13A	109.3
H9A—C9—H9B	109.6	C14—C13—H13A	109.3
N2—C9—H9C	110.5	N1—C13—H13B	109.3
C10'—C9—H9C	110.5	C14—C13—H13B	109.3
C10—C9—H9C	105.6	H13A—C13—H13B	108.0
H9B—C9—H9C	116.1	C15—C14—C13	124.2 (3)
N2—C9—H9D	110.5	C15—C14—H14	117.9
C10'—C9—H9D	110.5	C13—C14—H14	117.9
C10—C9—H9D	121.5	C14—C15—H15A	120.0
H9A—C9—H9D	101.8	C14—C15—H15B	120.0
H9C—C9—H9D	108.6	H15A—C15—H15B	120.0
C11—C10—C9	104.3 (14)		
			
C4—N1—C1—O1	−180.0 (2)	C2—N2—C9—C10	150.1 (10)
C13—N1—C1—O1	7.7 (3)	C12—N2—C9—C10	−21.4 (10)
C4—N1—C1—C12	−2.2 (3)	N2—C9—C10—C11	41.2 (14)
C13—N1—C1—C12	−174.50 (19)	C10'—C9—C10—C11	−87 (8)
C9—N2—C2—O2	6.1 (3)	C9—C10—C11—C12	−45.3 (16)
C12—N2—C2—O2	176.6 (2)	N2—C9—C10'—C11'	11.4 (17)
C9—N2—C2—C3	−169.74 (19)	C10—C9—C10'—C11'	65 (8)
C12—N2—C2—C3	0.8 (3)	C9—C10'—C11'—C12	−6.8 (19)
O2—C2—C3—C8	−35.3 (3)	C2—N2—C12—C1	70.3 (3)
N2—C2—C3—C8	140.6 (2)	C9—N2—C12—C1	−118.3 (2)
O2—C2—C3—C4	146.2 (2)	C2—N2—C12—C11'	−163.4 (7)
N2—C2—C3—C4	−38.0 (3)	C9—N2—C12—C11'	7.9 (7)
C8—C3—C4—C5	−1.9 (3)	C2—N2—C12—C11	−177.1 (6)
C2—C3—C4—C5	176.60 (19)	C9—N2—C12—C11	−5.7 (6)
C8—C3—C4—N1	173.72 (18)	O1—C1—C12—N2	107.3 (3)
C2—C3—C4—N1	−7.8 (3)	N1—C1—C12—N2	−70.5 (3)
C1—N1—C4—C5	−134.8 (2)	O1—C1—C12—C11'	−10.4 (9)
C13—N1—C4—C5	37.4 (3)	N1—C1—C12—C11'	171.8 (8)
C1—N1—C4—C3	49.6 (3)	O1—C1—C12—C11	−2.6 (8)
C13—N1—C4—C3	−138.2 (2)	N1—C1—C12—C11	179.7 (8)
C3—C4—C5—C6	−0.7 (3)	C10'—C11'—C12—N2	−0.5 (14)
N1—C4—C5—C6	−176.43 (19)	C10'—C11'—C12—C1	119.3 (11)
C4—C5—C6—C7	2.4 (3)	C10'—C11'—C12—C11	85 (7)
C5—C6—C7—C8	−1.4 (4)	C10—C11—C12—N2	30.4 (12)
C6—C7—C8—C3	−1.2 (4)	C10—C11—C12—C1	144.0 (10)
C4—C3—C8—C7	2.9 (3)	C10—C11—C12—C11'	−68 (6)
C2—C3—C8—C7	−175.8 (2)	C1—N1—C13—C14	73.4 (3)
C2—N2—C9—C10'	159.3 (10)	C4—N1—C13—C14	−99.4 (3)
C12—N2—C9—C10'	−12.3 (10)	N1—C13—C14—C15	139.5 (3)
